# A model for classification of invasive fungal rhinosinusitis by computed tomography

**DOI:** 10.1038/s41598-020-69446-5

**Published:** 2020-07-28

**Authors:** Guy Slonimsky, Johnathan D. McGinn, Neerav Goyal, Henry Crist, Max Hennessy, Eric Gagnon, Einat Slonimsky

**Affiliations:** 1 Department of Otolaryngology-Head and Neck Surgery, College of Medicine, The Pennsylvania State University, Hershey, PA USA; 2 Department of Pathology, College of Medicine, The Pennsylvania State University, Hershey, PA USA; 3 Department of Diagnostic Radiology, College of Medicine, The Pennsylvania State University, 500 University Drive, MB CG533, P.O. Box 850, Hershey, PA 17033-0850 USA

**Keywords:** Microbiology, Oncology, Risk factors

## Abstract

Our purpose was to classify acute invasive fungal rhinosinusitis (AIFR) caused by *Mucor* versus *Aspergillus* species by evaluating computed tomography radiological findings. Two blinded readers retrospectively graded radiological abnormalities of the craniofacial region observed on craniofacial CT examinations obtained during initial evaluation of 38 patients with eventually pathology-proven AIFR (13:25, *Mucor*:*Aspergillus*). Binomial logistic regression was used to analyze correlation between variables and type of fungi. Score-based models were implemented for analyzing differences in laterality of findings, including the ‘unilateral presence’ and ‘bilateral mean’ models. Binary logistic regression was used, with Score as the only predictor and Group (*Mucor* vs *Aspergillus*) as the only outcome. Specificity, sensitivity, positive predictive value, negative predictive value and accuracy were determined for the evaluated models. Given the low predictive value of any single evaluated anatomical site, a ‘bilateral mean’ score-based model including the nasal cavity, maxillary sinuses, ethmoid air cells, sphenoid sinus and frontal sinuses yielded the highest prediction accuracy, with *Mucor* induced AIFR correlating with higher prevalence of bilateral findings. The odds ratio for the model while integrating the above anatomical sites was 12.3 (*p* < 0.001). PPV, NPV, sensitivity, specificity and accuracy were 0.85, 0.82, 0.92, 0.69 and 0.84 respectively. The abnormal radiological findings on craniofacial CT scans of *Mucor* and *Aspergillus* induced AIFR could be differentiated based on laterality, with *Mucor* induced AIFR associated with higher prevalence of bilateral findings.

## Introduction

Acute invasive fungal rhinosinusitis (AIFR) is a rapidly progressive and life-threatening infection involving the nasal cavity and paranasal sinuses^1–3^. Patients with early stage AIFR limited to the nasal cavity and paranasal sinuses, have relatively lower mortality rates^2^, while intracranial extension doubles the mortality^4^. While a variety of causative organisms have been identified, *Aspergillus* and *Mucor* fungal species are predominant^3^. The most commonly predisposing conditions involve immunodeficiency and include hematologic malignancies, poorly controlled diabetes mellitus, chemotherapy or immunosuppression due to hematopoietic stem cells or organ transplantation^5,6^. Even though AIFR is a rare disease, its high mortality rate of approximately 50%^7^ highlights the importance of an appropriate and early diagnosis followed by aggressive treatment utilizing a combination of surgical debridement, antifungal pharmacotherapy and restoration of the patient's immune system when possible^8^. In many cases, AIFR is a manifestation of an overall poor prognosis with mortality attributed to the underlying medical condition^3^.

Early diagnosis and treatment of AIFR is of paramount importance to reduce patient morbidity and mortality. Effective treatment consists of an early and aggressive debridement of the necrotic tissue to decrease the fungal load and reduce impediments (e.g. vascular thrombosis) to antifungal delivery to remaining viable tissue, along with antifungal therapy and reconstitution of the patient's immune system^9–17^. Due to the importance of rapid treatment initiation, empiric antifungal pharmacotherapy might be initiated when AIFR is suspected, and will be further modified, as needed, according to pathology results.

Craniofacial computed tomography (CT) is a valuable tool in the early evaluation and for surgical planning for patients with AIFR despite its low sensitivity and specificity^18–20^. In many cases, imaging is obtained prior to the consultation with an otolaryngologist and nasal endoscopy. The most common CT findings of early AIFR include sinonasal mucosal thickening, air/fluid levels, soft-tissue infiltration of the maxillary periantral fat planes, infiltration of the middle turbinate and sinus opacification^1,2,18,21,22^. Radiological findings of advanced disease include bony dehiscence, orbital invasion, and intracranial extension^1,18,19,23,24^.

To the best our knowledge, the differences between the radiological findings in CT scans of patients with AIFR induced by *Mucor* versus *Aspergillus* were not evaluated so far. In the present study, we aimed to detect the possible differences of the imaging abnormalities found on craniofacial CT obtained during initial evaluation of patients eventually diagnosed with AIFR caused by *Mucor* and *Aspergillus* species.

## Materials and methods

### Patient selection and study design

The study was approved by the institutional review board at the Pennsylvania State University, College of Medicine. Institutional review board that approved the study waived the need for informed consent as part of the study approval. All procedures contributing to this work complied with the ethical standards of the relevant national and institutional guidelines on human experimentation, the human subjects protection office, and with the Helsinki Declaration of 1975, as revised in 2008.

Patients with nasal and paranasal sinus biopsies between January 1, 2007 and December 31, 2017 that were positive for invasive mucormycosis and invasive asperguillosis were included in this study. Inclusion criteria were: (1) Histopathologically proven invasive fungal rhinosinusitis with positive culture growth of either *Mucor* or *Aspergillus* species, and (2) CT imaging of the craniofacial region within 5 days preceding tissue biopsy. Subjects were excluded if the CT imaging was inadequate due to: (1) partial coverage of the nasal cavity, paranasal sinuses, orbits or intracranial space, or (2) the presence of severe dental artifacts impeding analysis of the imaging.

Clinical data was collected from the medical records including demographic data, surgical and histopathology reports, culture results, underlying medical conditions, antifungal treatment, absolute neutrophil count (ANC) and outcome. Outcome was classified as either deceased from AIFR, deceased not from AIFR, not deceased, and uncertain.

### Imaging protocol

All studies were performed in our institution, using the institutional craniofacial CT protocol. According to our protocol, images were acquired as 1–3-mm-thick sections and an in-plane FOV from 170 to 190 mm. Soft tissue and bone algorithm reconstructions were performed.

Scans were obtained in an axial plane and included the paranasal sinuses and hard palate. Multiplanar reformations were obtained in the coronal and sagittal plane. None of the patients in the *Aspergillus* group received contrast while three subjects from the *Mucor* group had contrast enhanced CT scans.

### Image analysis

Studies were reviewed independently by two readers, a neuroradiologist and an otolaryngologist, both blinded to the patients' clinical information and histopathology results. There was consensus between the readers. The readers evaluated and graded as 'present' or 'absent' mucosal thickening or infiltration in the following anatomical sites: anterior periantral fat (soft tissue anterior to the anterior wall of the maxillary sinus), posterior periantral fat, sphenopalatine foramen, pterygopalatine fossa, nasolacrimal duct, medial orbital fat,inferior orbital fat and facial soft tissue including other location not specified, including but not limiting to, the masticator space. Mucosal thickening of the nasal cavity was defined as more than 3 mm and was recorded as present or absent. Bony dehiscence of the sinonasal area or hard palate were recorded as present or absent.

Sinus opacification was graded according to the amount of mucosal thickening in the paranasal sinuses (maxillary sinuses, frontal sinuses, sphenoid sinuses and ethmoid air cells) on a scale of 0–3 (0; no mucosal thickening, 1; < 50% opacified, 2; > 50% opacified, 3; 100% opacified).

### Statistical analysis

The R statistical programing language (version 3.4.4, 2018–03-15) was used to clean, transform, and analyze the results. Pearson’s correlation coefficient (ρ), Kendall’s correlation coefficient (τ), and Cramer’s V coefficient were used to analyze pairwise relationships between variables, both for identifying potential confounders and in guiding inclusion in models. Several modeling techniques were attempted including binomial logistic regression and Canonical correlation analysis.

A score-based model was implemented that included multiple factors, based on methodology by Middlebrooks et al.^20^. Two techniques for analyzing differences in laterality of findings were used to construct scores: the “unilateral presence” model assigns a value of one for a unilateral presence of a factor, and uses the lateral mean for each factor as its value, and the “bilateral mean” model scales each factor’s possible values between zero and one and uses the lateral mean for each factor as its value.

Values for each technique were summed to construct a score. Binary logistic regression was used, with Score as the only predictor and Group (*Mucor* vs *Aspergillus*) as the only outcome. Results were considered significant with α ≤ 0.05.

### Ethical approval

All procedures performed in the study involving human participants were in accordance with the ethical standards of the institutional and/or national research committee and with the 1964 Helsinski Declaration and its later amendments or comparable ethical standards.

### Informed consent

Informed consent was waived from all individual participants included in the study.

## Results

Thirty-eight patients met the inclusion criteria, thirteen patients (34.2%) diagnosed with invasive mucormycosis and twenty-five patients (65.8%) with invasive aspergillosis. Table 1 describes the demographic and clinical variables collected. There was no difference in the demographic characteristics between the two groups. The most prevalent predisposing condition was AML for both *Mucor* (76.9%) and *Aspergillus* (56%) patients without any statistically significant differences between the distributions of conditions. There was no significant difference in the ANC for both groups. One patient had an ANC > 500. This patient had poorly controlled diabetes without an underlying hematological malignancy.

**Table 1:** Demographics and clinical characteristics of both patient groups.

Characteristics	*Mucor*	*Aspergillus*	*p* value
Mean age, years (SD)	52.5 (12.5)	51.6 (17.2)	0.979
M:F	7:6	14:11	0.468
ANC^a^ < 500	10/13	23/25	
ANC > 500	0/13	1/25	
ANC unknown	3/13	1/25	
Outcome			0.223
AIFR-related mortality	7	9	
Non-AIFR^b^ related mortality	1	8	
Not deceased	3	7	
Condition			0.292
AML^c^	10	14	
ALL^d^	2	3	
MDS^e^	0	3	
MM^f^	1	1	
Others	0	4	

^a^ANC, absolute neutrophil count; ^b^AIFR, acute invasive fungal rhinosinusitis; ^c^AML, acute myeloid leukemia; ^d^ALL, acute lymphocytic leukemia; ^e^MDS, myelodysplastic syndrome; ^f^MM, multiple myeloma.

Severe soft tissue thickening, including the turbinates, nasal walls, septum, and anterior and posterior periantral fat was present in 5 of the 13 *Mucor* patients in this study and in 9 of the 25 *Aspergillus* patients in this study. Severe orbital soft tissue thickening and fatty standing was present in 3 of the 13 *Mucor* patients in this study and in 5 of the 9 *Aspergillus* patients in this study. Sinus mucosal thickening was present in 13 out of 13 *Mucor* patients and in 24 out of 25 *Aspergillus* patients in this study.

**Figure 1:**
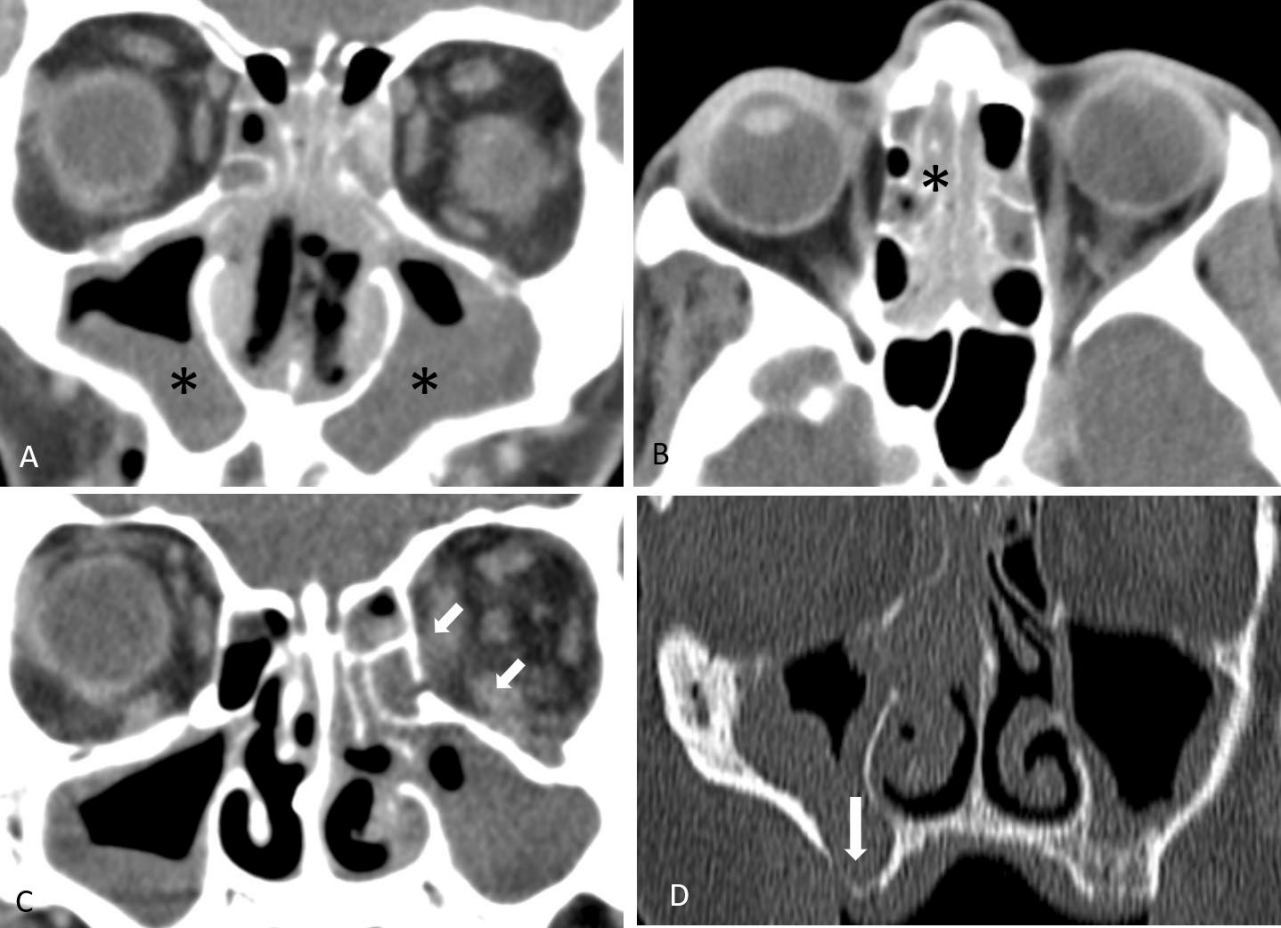
Examples of findings in patients with *Mucor* AIFR. (**A**) Coronal CT image shows bilateral mucosal thickening involving the maxillary sinuses (asterisks) and the nasal cavity. (**B**) Axial image in the same patient demonstrates bilateral mucosal thickening of ethmoid air cells (asterisk). (**C**) Coronal image in a different patient shows bilateral maxillary sinuses involvement and left orbital involvement with fatty infiltration of the left medial and inferior extraconal orbital fat (arrows). (**D**) Coronal CT of a different patient shows bilateral mucosal thickening of the maxillary sinuses with bony dehiscence along the inferior aspect of the right maxillary sinus.

**Figure 2:**
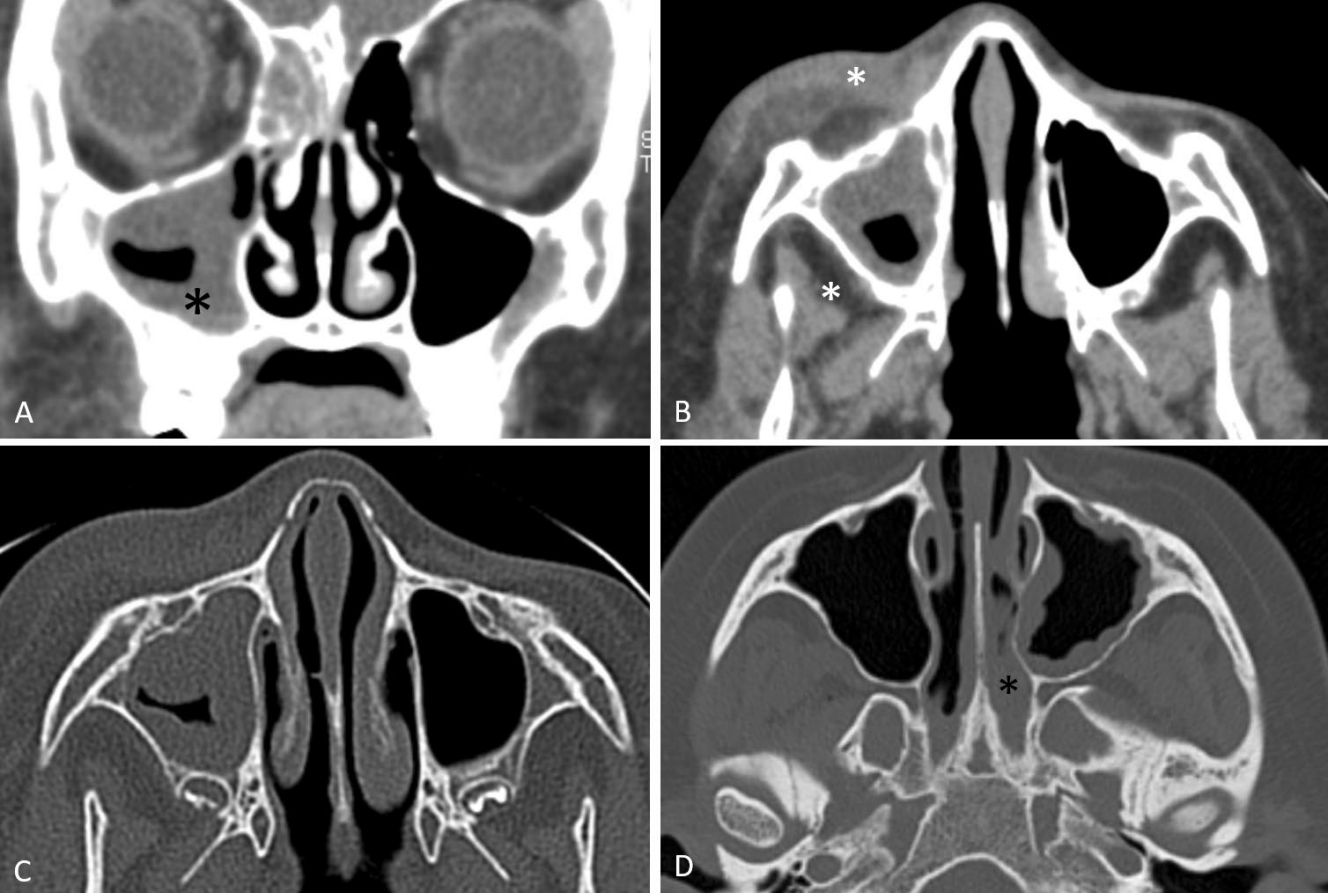
Examples of findings in patients with *Aspergillus* AIFR. (**A**) Coronal CT image shows unilateral mucosal thickening of the right maxillary sinus (asterisk) and the ethmoid air cells. (**B**, **C**) Axial images from the same patient shows fatty infiltration of the anterior periantral fat and the posterior periantral fat, without bony dehiscence. (**D**) Another patient with Aspergillus AIFR with unilateral mucosal thickening of the maxillary sinus and the nasal cavity (asterisk).

The average interval between CT examination and surgical intervention was 0.92 days (range 0–3 days) for *Mucor* patients and 1.1 days (range 0–5 days) for *Aspergillus* patients. By reviewing radiological abnormalities distribution for each of the aforementioned anatomical regions, no individual region or radiological pattern significantly correlated with either *Mucor* or *Aspergillus* AIFR. Hence, score-based models evaluating the laterality of findings from each of the aforementioned anatomical regions were applied. Examples for abnormal radiological findings in patients with *Mucor* and *Aspergillus* are presented in Figs. 1 and 2.

### Score based models

Unilateral presence and bilateral mean score-based models were applied for the data collected from each of the aforementioned anatomical regions.

Unilateral presence, though close, did not reach statistical significance and yielded lower PPV, NPV, sensitivity, specificity and accuracy and thus was not selected. The bilateral mean model was tested with various combinations of the evaluated anatomical regions, yielded statistically significant results and was found to be the most predictive model to classify between *Mucor* and *Aspergillus* AIFR.

Furthermore, the scores for the bilateral mean model yielded the highest accuracy when data from the following anatomical regions, as well as the presence of bony dehiscence, were included:

- Nasal cavity
- Maxillary sinuses
- Ethmoid air cells
- Sphenoid sinus
- Frontal sinuses
- Bony dehiscence

The odds ratio for the model while integrating the above anatomical sites was 12.3 (*p* < 0.001). PPV, NPV, sensitivity, specificity and accuracy were 0.85, 0.82, 0.92, 0.69 and 0.84 respectively. The addition of orbital involvement to the data resulted in a statistically significant model although with slightly reduced PPV, NPV, sensitivity and accuracy, and therefore was not included (Table 2).

**Table 2:** AIFR classification of *Aspergillus* versus *Mucor* in various combinations of the bilateral mean model.

Item	Bilateral mean^a^	Bilateral mean with bony dehiscence^b^	Bilateral mean with orbits^c^	Bilateral mean with orbits and bony dehiscence^d^
Effect from score on odds for Mucor	12.43	12.30	5.27	6.45
*p* value for effect from score	0.01	0.00	0.01	0.00
PPV: Mucor	0.81	0.85	0.76	0.83
NPV: Mucor	0.80	0.82	0.58	0.69
Sensitivity: Mucor	0.92	0.92	0.79	0.83
Specificity: Mucor	0.62	0.69	0.54	0.69
Accuracy: Mucor	0.81	0.84	0.70	0.78

^a^Model included nasal cavity, maxillary sinus, ethmoid air cells, sphenoid sinus and frontal sinus.

^b^Model included nasal cavity, maxillary sinus, ethmoid air cells, sphenoid sinus, frontal sinus and bony dehiscence.

^c^Model included nasal cavity, maxillary sinus, ethmoid air cells, sphenoid sinus, frontal sinus and orbital involvement.

^d^Model included nasal cavity, maxillary sinus, ethmoid air cells, sphenoid sinus, frontal sinus, bony dehiscence and orbital involvement.

### Cutoff score

**Figure 3:**
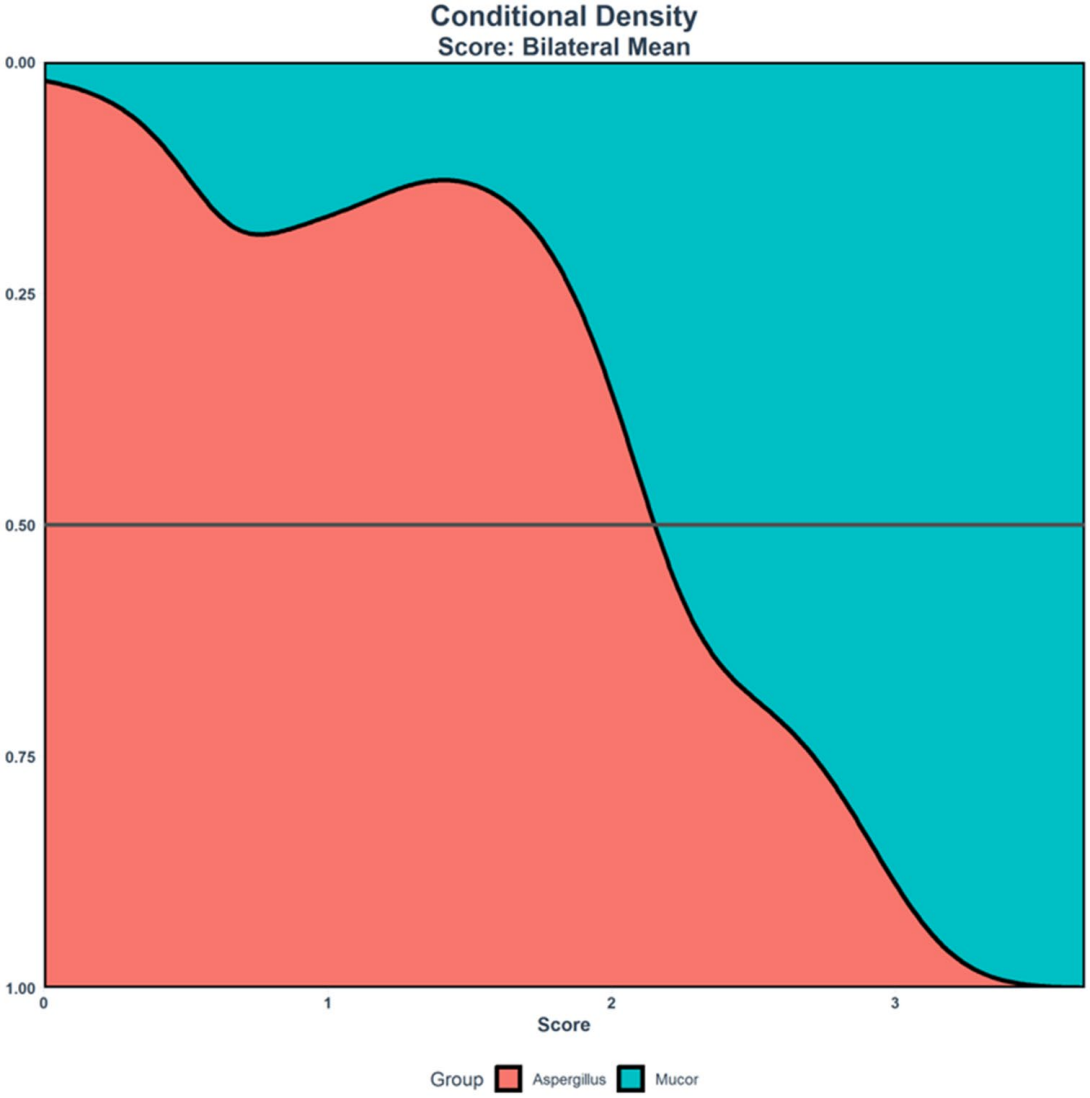
Conditional density diagram of the bilateral mean model. Patients with AIFR caused by *Mucor* species had higher mean score values compared with patients with AIFR caused by *Aspergillus* species.

The cutoff (transition) score to predict *Mucor* versus *Aspergillus* AIFR was calculated for the bilateral mean model. Scores above the cuttoff are more likely to be *Mucor* than *Aspergillus* (Table 3). The cut off score at 50% for the bilateral mean model was 2.15. This can also be appreciated by the conditional density diagram (Fig. 3).

**Table 3:** Cutoffs for scores for predicting *Aspergillus* versus *Mucor* in various combinations of the bilateral mean model.

Model	Threshold score	Probability of *Mucor* at threshold
Bilateral mean^a^	2.06	0.51
Bilateral mean with bony dehiscence^b^	2.15	0.5
Bilateral mean with orbits^c^	2.34	0.5
Bilateral mean with orbits and bony dehiscence^d^	2.43	0.5

^a^Model included nasal cavity, maxillary sinus, ethmoid air cells, sphenoid sinus and frontal sinus.

^b^Model included nasal cavity, maxillary sinus, ethmoid air cells, sphenoid sinus, frontal sinus and bony dehiscence.

^c^Model included nasal cavity, maxillary sinus, ethmoid air cells, sphenoid sinus, frontal sinus and orbital involvement.

^d^Model included nasal cavity, maxillary sinus, ethmoid air cells, sphenoid sinus, frontal sinus, bony dehiscence and orbital involvement.

## Discussion

We retrospectively evaluate the presence and distribution of radiological abnormalities in patients with histopathologically proven *Mucor* or *Aspergillus* induced AIFR. Since *Mucor* and *Aspergillus* species constitute the vast majority of the offending organisms in AIFR, other rare types of fungi were not included in our study.

Our cohort included a total of 38 patients. Thirteen patients with invasive mucormycosis and twenty-five patients with invasive aspergillosis. By incorporating various abnormal radiological craniofacial CT findings from specific anatomical regions into the bilateral mean model, we found that AIFR caused by *Mucor* species demonstrated a higher degree of bilateral sinonasal involvement. Furthermore, as the calculated score rose above the cutoff score (more bilateral findings), the probability for *Mucor* (vs *Aspergillus*) increased. The application of this model demonstrated the radiological differences in the laterality of findings between *Aspergillus* and *Mucor* AIFR.

Identifying radiological differences for AIFR induced by *Mucor* versus *Aspergillus* species on craniofacial CT scans, has the potential to predict the inciting pathogen and potentially facilitate guidance of a more targeted pharmacological treatment prior to definite tissue diagnosis in patients with a high index of suspicion for AIFR.

Interestingly, previous studies found unilateral mucosal thickening and soft tissue infiltration to be associated with AIFR^1^. Middlebrooks et al.^20^ found that 78.6% of their patients had unilateral predominant findings. Those studies evaluated cohorts of patients with AIFR induced by various fungi species but did not contrast these findings in *Mucor* versus *Aspergillus* species. Therefore, as *Aspergillus* is a considerably more prevalent pathogen in AIFR compared to *Mucor,* it could possibly shift the pendulum towards unilaterality of the abnormal CT findings, which is in line with our results that demonstrated a higher likelihood of unilateral findings with *Aspergillus*.

Tissue biopsy is the gold standard for diagnosing and classifying the pathogen in AIFR, with histology demonstrating fungal invasion. It mandates intranasal endoscopy performed either at bedside or in the operating room. In most cases, AIFR is the hallmark of an overall severely deteriorated medical state which may include severe thrombocytopenia and even hemodynamic instability which may delay obtaining tissue biopsy.

Craniofacial CT scan will typically be the imaging modality of choice for the initial patient's evaluation, while MRI is usually reserved for cases of clinically suspected intracranial, base of skull or orbital involvement. Bony dehiscence is a rare and insensitive radiological finding that is usually detected in cases of very advanced disease^1,20,24,26^. Generally, craniofacial CT will demonstrate nonspecific findings similar to acute or chronic rhinosinusitis^1,18,21,26^. Finkelstein et al.^26^ compared craniofacial CT abnormalities of 14 patients with AIFR (8 patients with *Mucor* and 6 with *Aspergillus* species), with those of 20 patients with suspected, but finally excluded, AIFR. Thirteen imaging parameters were evaluated from which bony dehiscence, facial soft tissue thickening, extra sinus extension and unilaterality were statistically associated with AIFR. Nevertheless, they found that craniofacial CT findings on the early stages of AIFR are nonspecific and must be complemented with additional diagnostic tools. In a retrospective case/control study, DelGuadio et al.^18^ compared CT findings of 23 patients with AIFR (9 patients with *Mucor* and 14 with *Aspergillus* species) with those of 10 control patients with acute myelocytic leukemia (AML) and nonfungal rhinosinusitis. They found severe unilateral nasal mucosal thickening to be the most consistent finding in AIFR. Middlebrooks et al.^20^ analyzed 23 variables of CT findings from 42 patients with AIFR (10 patients with *Mucor*, 18 with *Aspergillus*, and the rest with various fungal species) versus 42 control patients proved negative for AIFR, in order to design a diagnostic imaging model. By multivariate analysis, the group finally developed a model consisting of 7 variables including bony dehiscence, orbital invasion, and septal ulceration, involvement of periantral fat, pterygopalatine fossa, nasolacrimal duct and lacrimal sac. The presence of an abnormality of any additional variable increased the positive/negative predictive value, sensitivity and specificity regarding the diagnosis of AIFR. Bony dehiscence was reported to have 100% specificity and 35% sensitivity for AIFR. None of the aforementioned studies discriminated *Mucor* versus *Aspergillus* species in their data analysis.

Our results demonstrate that while AIFR caused by *Mucor* and *Aspergillus* share overall similar abnormal findings on craniofacial CT, the two pathogens may be differentiated by laterality of radiological findings as demonstrated by the bilateral mean model.

The limitations of our study include a retrospective study, a limited cohort of patients and the exclusion of fungi species other than *Mucor* and *Aspergillus*. These limitations are derived from the fact that AIFR is rare, and although this is one of the largest cohorts of AIFR patients reported, the power of statistical analysis is limited.

In this study we demonstrated that although the abnormal radiological finding on craniofacial CT scans of *Mucor* and *Aspergillus* induced AIFR are similar, they could be differentiated based on laterality. By using the bilateral mean model, we demonstrated that Mucor induced AIFR is associated with higher prevalence of bilateral findings. Similar to prior studies evaluating patients with AIFR, facial soft tissue thickening and orbital involvement were not common features^4,25^. Mucosal thickening in the sinuses was a common feature both in *Mucor* and *Aspergillus* AIFR as has been previously reported^18,20,26^.

It is important to highlight that our model does not intend to make a diagnosis of *Mucor* versus *Aspergillus* AIFR based on imaging findings solely. The workflow should be imaging findings that raise concern of AIFR specifically in at risk population presenting with relevant clinical symptoms and with suspicious finding on physical examination/nasal endoscopy concerning for AIFR, and then apply the model to further predict *Mucor* versus *Aspergillus* (as most likely pathogen) while waiting for final pathological results. Besides a potential clinical significance, we believe that this model provides a better understanding of the radiological manifestation of AIFR specifically understanding the subtle differences between *Mucor* and *Aspergillus* inflicted disease.

To the best of our knowledge, this is the first study to directly compare radiological finding of patients with *Mucor* versus *Aspergillus* induced AIFR. We believe that our findings could potentially contribute to future studies incorporating the rapidly evolving machine learning algorithms for better classification of diseases by imaging.

## Data Availability

The authors declare to make materials, data and associated protocols promptly available to readers without undue qualifications.
